# Regulation of Isoleucine on Colonic Barrier Function in Rotavirus-Infected Weanling Piglets and Analysis of Gut Microbiota and Metabolomics

**DOI:** 10.3390/microorganisms12122396

**Published:** 2024-11-22

**Authors:** Changsheng Jiang, Weiying Chen, Yanan Yang, Xiaojin Li, Mengmeng Jin, Ahmed H. Ghonaim, Shenghe Li, Man Ren

**Affiliations:** 1 Anhui Provincial Key Laboratory of Animal Nutritional Regulation and Health, College of Animal Science, Anhui Science and Technology University, Chuzhou 233100, China; jiangcs@ahstu.edu.cn (C.J.); 18010919050@163.com (W.C.); 17855022027@163.com (Y.Y.); lixj@ahstu.edu.cn (X.L.); jinmm@ahstu.edu.cn (M.J.); 2 National Key Laboratory of Agricultural Microbiology, College of Animal Sciences and Veterinary Medicine, Huazhong Agricultural University, Wuhan 430070, China; a.ghonaim@webmail.hzau.edu.cn; 3 Desert Research Center, Cairo 11435, Egypt

**Keywords:** isoleucine, intestinal barrier, rotavirus, piglets, gut microbiota, metabolomics

## Abstract

Rotavirus (RV) is a significant contributor to diarrhea in both young children and animals, especially in piglets, resulting in considerable economic impacts on the global pig industry. Isoleucine (Ile), a branched-chain amino acid, is crucial for regulating nutrient metabolism and has been found to help mitigate diarrhea. This study aimed to assess the impact of isoleucine supplementation in feed on colonic barrier function, colonic microbiota, and metabolism in RV-infected weanling piglets. A total of thirty-two weaned piglets, aged 21 days, were randomly assigned to two dietary groups (each further divided into two subgroups, with eight replicates in each subgroup), receiving diets with either 0% or 1% isoleucine for a duration of 14 days. One group from each treatment was then challenged with RV, and the experimental period lasted for 19 days. The results showed that dietary Ile significantly increased the secretion of IL-4, IL-10, and sIgA in the colon of RV-infected weanling piglets (*p* < 0.05). In addition, Ile supplementation notably increased the expression of tight junction proteins, including Claudin-3, Occludin, and ZO-1 (*p* < 0.01), as well as the mucin protein MUC-1 in the colon of RV-infected weanling piglets (*p* < 0.05). Gut microbiota analysis revealed that dietary Ile increased the relative abundance of Prevotella and decreased the relative abundance of Rikenellaceae in the colons of RV-infected weanling piglets. Compared with the RV+CON, metabolic pathways in the RV+ILE group were significantly enriched in vitamin digestion and absorption, steroid biosynthesis, purine metabolism, pantothenate and CoA biosynthesis, cutin, suberine, and wax biosynthesis, as well as fatty acid biosynthesis, and unsaturated fatty acid biosynthesis. In conclusion, dietary Ile supplementation can improve immunity, colonic barrier function, colonic microbiota, and colonic metabolism of RV-infected weaned piglets. These findings provide valuable insights into the role of isoleucine in the prevention and control of RV.

## 1. Introduction

Rotavirus (RV) is a zoonotic pathogen that causes acute enteritis in infants and animals, including suckling piglets and weaned piglets, which is characterized by fever, diarrhea, and dehydration [1,2,3]. The incomplete development of the immune and digestive systems of weaned piglets leads to higher morbidity and mortality rates, with a morbidity rate of 80% and a mortality rate of 20%, causing huge economic losses to the global pig industry [4]. Intestinal epithelial cells (IECs) serve as the primary barrier against RV infection. RV infection can damage intestinal epithelial cells, then result in villus atrophy, apoptosis of IECs, eventually leading to diarrhea [5]. Previous studies have shown that the susceptible site of RV is mainly located in the small intestine, where the virus directly damages the villous epithelial cells, leading to cell destruction and shedding [5,6,7]. This destruction results in the accumulation of lactose in the intestinal lumen, creating high osmotic pressure in both the small and large intestine, which in turn causes diarrhea and vomiting [8]. The damage to intestinal cells impairs the epithelial barrier, leading to a loss of digestive and absorptive functions, which can accelerate the death of piglets [9]. A previous study confirmed that RV can infect and replicate in the colon of humans and mice [10]. However, it is not fully understood whether RV affects the intestinal barrier of the colon in piglets. Therefore, this study aims to investigate the role of the colonic intestinal barrier in resisting RV infection.

The intestinal barrier, composed of intestinal epithelial cells, tight junctions, related secretions, and intestinal microbiota, serves as a crucial defense mechanism against the invasion of pathogenic microorganisms into the internal environment of the body [11]. In weaned piglets, intestinal barrier dysfunction is often observed, accompanied by increased permeability, which subsequently leads to diarrhea and growth retardation [6]. Therefore, effective and safe methods need to be developed to maintain the intestinal barrier function in piglets to resist pathogen invasion.

Previous studies have shown that amino acids in the diet can significantly impact intestinal health [12,13,14]. Isoleucine (Ile) is one of the essential amino acids in animals, serves as a regulatory factor for the three major nutritional metabolism and conversion processes. It acts as an energy source for the immune system and a substrate for the synthesis of immune proteins [15]. Studies have shown that isoleucine can improve the growth performance of weaned piglets on protein-restricted diets [16,17,18]. Additional isoleucine supplementation promotes intestinal development in weaned piglets, increases immunoglobulin concentrations in the jejunal mucosa, and reduces the expression of pro-inflammatory cytokines [19]. However, it remains unclear whether isoleucine can regulate intestinal barrier function. Therefore, this study aims to investigate the effects of isoleucine on colonic barrier function in RV-infected weaned piglets, as well as the changes in colonic fecal microbiota, to explore its mechanism on intestinal barrier function. These findings will provide a theoretical basis for the role of isoleucine in disease resistance and prevention.

## 2. Materials and Methods

### 2.1. Virus Culture

MA104 cells (ATCC CRL-2378), derived from African green monkey kidney cells, were purchased from the American type culture collection (ATCC). The MA104 cells were cultured in Dulbecco’s modified eagle medium (DMEM, Gibco, Waltham, MA, USA) supplemented with 10% fetal bovine serum (FBS, Gibco) in a 37 °C incubator with 5% CO_2_. The cells were seeded in cell culture plates and incubated at 37 °C with 5% CO_2_. Once the cells reached 60% to 70% confluence, the cells were digested with 0.25% trypsin-EDTA (Merck, Burlington, MA, USA) and inoculated with RV virus (preserved at the National Veterinary Microbiological Culture Collection Management Center, Beijing, China). After two hours of incubation, the MEM medium without fetal bovine serum was replaced, and the cells were cultured for an additional 3 to 5 days before collecting the virus. This infection procedure was repeated with the collected virus until over 70% of the MA104 cells in the culture plate exhibited significant cytopathic effects within 3 days post inoculation [20]. The virus titer was then determined to be 10^6^ TCID_50_/mL, and the virus was frozen for subsequent challenge experiments.

### 2.2. Experimental Design

All animal experiments were approved by the Animal Ethics Committee of Anhui Science and Technology University, under protocol number AK2023013. All experimental procedures were carried out in strict accordance with the “Guidelines for the Care and Use of Test Animals” of Anhui Province.

**Figure 1:**
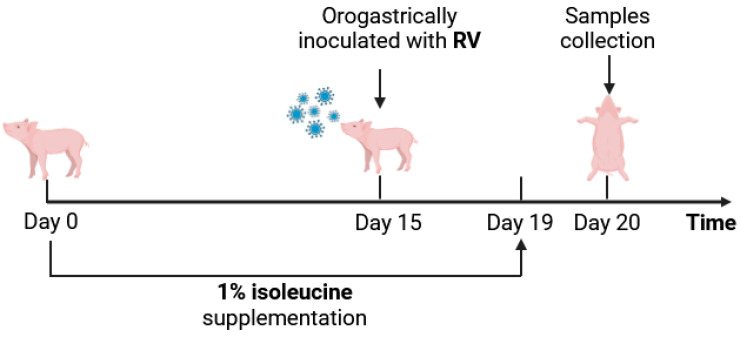
Timeline of the events in the experimental study.

Thirty-two healthy Duroc×Landrace×Yorkshire weaned barrows, with similar body weights (6.88 ± 0.54 kg) at 21-days of age, were randomly divided into four groups (eight replicates in each group). The RV-CON and RV+CON groups were fed a basal diet, while the RV-Ile and RV+ Ile received a basal diet supplemented with 1% Ile. On the 15th day of feeding, all piglets were orogastrically inoculated with 5 mL of 100 mmol/L sodium bicarbonate (NaHCO_3_) solution, and 30 min later, the infected group was orogastrically inoculated with 5 mL of RV (1 × 10^6^ TCID_50_/mL), while the uninfected group was orogastrically inoculated with 5 mL of saline. At 5 days post infection (dpi), all piglets were euthanized, and samples were collected. Approximately 2 cm of the colon was collected, and any fatty tissue on the surface of the intestine was carefully peeled off to avoid contamination during the sampling process. An additional 2 cm of fresh colon was collected and stored in liquid nitrogen, while another 2 cm was rinsed with saline and promptly fixed in 4% paraformaldehyde. The mucosa of colon was scraped into centrifuge tubes, frozen in liquid nitrogen, and subsequently stored at −80 °C. The animal experimental design and treatments are summarized in Table 1 and Figure 1.

### 2.3. Diet Composition

The basal diet was a corn–soybean meal formulation, designed according to the NRC (2012) guidelines (Table 2). The isoleucine nutritional level in the basal diet was set at 0.72%. For the different dietary groups, either 1% L-isoleucine (provided by Evonik Industries AG, Essen, Germany) or 1% L-alanine (Evonik Industries AG) was added, with alanine serving as the isonitrogenous control for isoleucine.

### 2.4. Tissue Staining

Fixed colonic tissues were subjected to a series of procedures, including gradient alcohol dehydration, clearing, wax impregnation, embedding, slicing, slide spreading, and baking, followed by hematoxylin and eosin (HE) staining according to the manufacturer’s protocol to assess tissue morphology. Subsequently, five visual fields were uniformly selected from each slice and photographed under the same magnification. The muscular layer thickness and crypt depth of the colon were measured using Image-Pro Plus (Media Cybernetics, Version 6.0, USA) microscopic image analysis software.

### 2.5. The Cytokines and sIgA Analysis

The levels of interleukin (IL)-2, IL-4, IL-10, IL-12, IL-18, IL-22, TNF-α, and sIgA in the colonic mucosa were determined using ELISA kits following the manufacturer’s instructions. All the ELISA kits were purchased from Shanghai Xinle Biotechnology Co., Ltd. (Shanghai, China) (Table 3).

### 2.6. Western Blot

The total protein of the colon was extracted using RIPA lysate supplemented with 1% PMSF (Beyotime, Shanghai, China) at 4 °C. The protein concentration was measured using a BCA Protein Assay Kit (Thermo Fisher, Waltham, MA, USA). A total of 20 μg of protein from each sample was loaded onto a 10% SDS-PAGE gel and then transferred to a polyvinylidene difluoride (PVDF) membrane. To prevent non-specific binding, the PVDF membranes were blocked with 5% bovine serum albumin (BSA) at room temperature for 2 h. Next, the membranes were incubated overnight at 4 °C with the appropriate primary antibody. Following this incubation, the membranes were washed three times with Tris-buffered saline containing Tween 20 (TBST), and then incubated with HRP-linked goat anti-rabbit antibodies at room temperature for 2 h. Visualization was performed using an ECL detection kit (Solarbio, Beijing, China). The antibodies used for Western blot are listed in Table 4.

### 2.7. Colonic Microbiota Analysis

Middle-section colonic digesta samples were collected and sent to Beijing Novogene Bioinformatics Technology Co., Ltd. (Beijing, China). DNA was extracted using a DNA extraction kit, and the V3-V4 region of 16S rRNA was targeted for PCR amplification. The primers were synthesized by Beijing Novogene Bioinformatics Technology Co., Ltd. Sequences from different samples were split using the OIIME2 demux plugin, and the split sequences underwent quality control, trimming, denoising, splicing, and chimera removal using the OIIME2 dada2 plugin to obtain the final feature sequences.

### 2.8. Colonic Contents Metabolomics Analysis

Samples were sent to Shanghai Majorbio Bio-pharm Technology Co., Ltd. (Shanghai, China) for GC-MS untargeted metabolomics analysis. The raw data were processed using the metabolomics processing software Progenesis QI 2.4 (Waters, Milford, MA, USA) to generate a data matrix. The mass spectrometry information was matched with the metabolic public database HMDB to obtain metabolite information. Differentially abundant metabolites were selected based on the criterion of *p* < 0.05 and VIP > 1, and volcano plots were generated using R language. The KEGG database was used for metabolic pathway annotation to identify pathways associated with differentially abundant metabolites, and enrichment analysis was performed.

### 2.9. Statistical Analysis

For the general data, the obtained results are expressed as the mean ± standard deviation (SD). Statistical analysis was performed using multiple *t*-tests in GraphPad Prism 6.0 (GraphPad Software Inc., USA). A *p* value of <0.05 was considered statistically significant (*), while *p* values of <0.01 (**) were considered highly significant. For microbiota and metabolomics data, the analysis was conducted using the free online platform provided by the sequencing company.

## 3. Results

### 3.1. Isoleucine Does Not Affect the Structure of the Colon in RV-Infected Weaning Piglets

**Figure 2:**
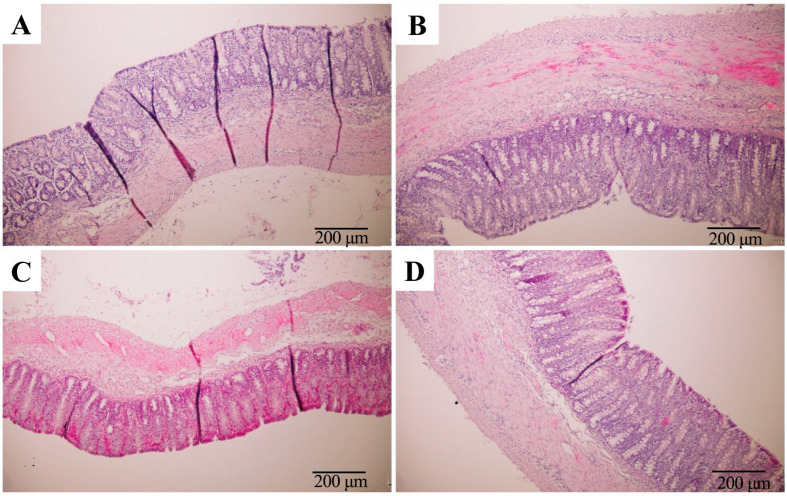
The histological structure of the colon in RV-infected weaned piglets (HE, 200×). (**A**) The RV-CON group, (**B**) the RV-Ile group, (**C**) the RV+CON group, and (**D**) the RV+Ile group. Bar = 200 μm.

To assess the potential impact of isoleucine on the colonic barrier function of piglets, the colonic structures were first observed. Compared to the small intestine, the colonic mucosal epithelium lacks villi, and the lamina propria contains numerous colonic glands, known as crypts, along with a well-developed muscular layer divided into inner circular, middle oblique, and outer longitudinal layers [21]. As shown in Figure 2 and Table 5, Ile did not affect the crypt depth or muscularis thickness of the colon in either RV infected or uninfected piglets. These results indicated that the addition of 1% isoleucine to the diet does not affect the structural integrity of the colon in RV-infected weaning piglets.

### 3.2. Isoleucine Improves the Immunity of the Colon in RV-Infected Piglets

To investigate whether isoleucine could alleviate intestinal inflammatory responses induced by rotavirus, cytokines in the colonic mucosa were measured using ELISA kits. As shown in Table 6, the addition of 1% isoleucine to the diet had no significant effect on the levels of IL-2, IL-12, IL-18, IL-22, and TNF-α in the colon of RV-infected or uninfected weaned piglets (*p* > 0.05). However, compared with the RV-CON group, 1% Ile significantly increased the concentration of IL-4 in the colonic mucosa of RV-uninfected piglets (*p* < 0.05). Additionally, compared with the RV+CON group, 1% Ile notably increased the concentration of IL-4 in the colonic mucosa of RV-infected piglets (*p* < 0.05). Although the concentration of IL-10 in the colonic mucosa of the RV+ILE group was higher than that in the RV+CON group, this difference was not statistically significant (*p* > 0.05). Since IL-4 and IL-10 are anti-inflammatory cytokines, these results suggested that the addition of 1% isoleucine to the diet may reduce the inflammatory responses in the colons of piglets caused by RV infection.

sIgA is a major immunoglobulin in the intestinal mucosal immune system and plays a crucial role in constituting the first line of defense in intestinal mucosal immunity. As shown in Table 6, the concentration of sIgA in the colonic mucosa was significantly increased in the RV+Ile group compared with the RV+CON group. This finding indicated that the addition of 1% isoleucine to the diet enhanced the mucosal immunity of the colon in RV-infected piglets. Taken together, these results demonstrated that the inclusion of 1% isoleucine in the diet improves the immunity of the colon in RV-infected piglets.

### 3.3. Isoleucine Improves the Intestinal Barrier of the Colon in RV-Infected Piglets

**Figure 3:**
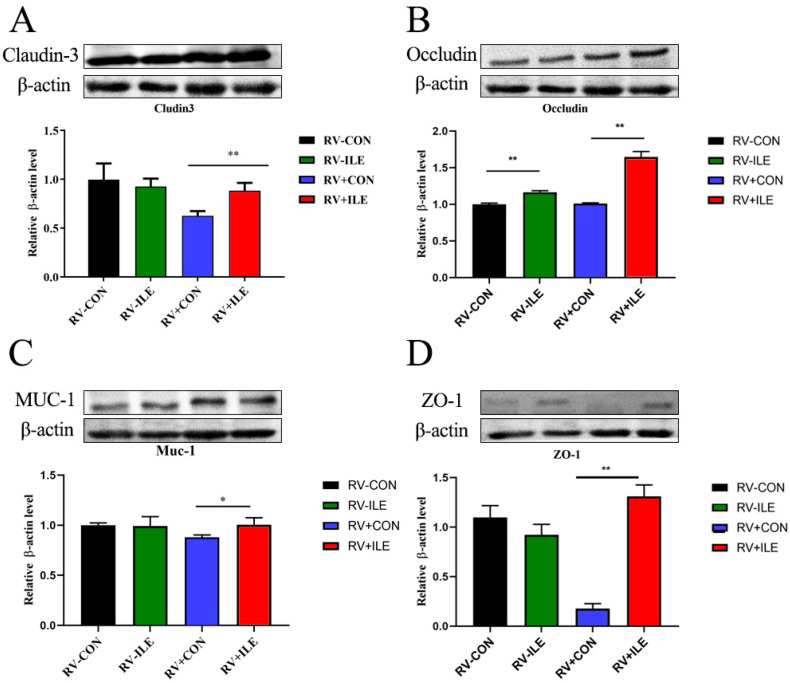
Effect of isoleucine on the expression of colonic tight junction protein, defensin and mucin protein in weaned Piglets. The proteins expression of Claudin 3 (**A**), Occludin (**B**), MUC-1 (**C**) and ZO-1 (**D**), respectively, in colon of RV-infected piglets. Data are presented as the mean ± S.D, * *p* < 0.05; ** *p* < 0.01.

To elucidate the protective mechanisms of isoleucine in mitigating intestinal inflammation, we examined the expression of tight junction proteins, including Claudin-3, Occludin, ZO-1, and MUC-1 in the colon of piglets. In RV-uninfected piglets, the addition of 1% isoleucine (Ile) did not change the expression levels of Claudin-3, MUC-1, or ZO-1 when compared to the RV-CON group (*p* > 0.05). However, there was a significant increase in Occludin expression in the colon of RV-uninfected piglets receiving 1% Ile compared to the RV-CON group (*p* < 0.05). Importantly, in RV-infected piglets, the inclusion of 1% Ile markedly elevated the expression levels of Claudin-3, Occludin, MUC-1, and ZO-1 compared to the RV+CON group (*p* < 0.05) (Figure 3). These findings suggested that isoleucine can promote the intestinal barrier function of the colon in RV-infected piglets.

### 3.4. Gut Microbiota

**Figure 4:**
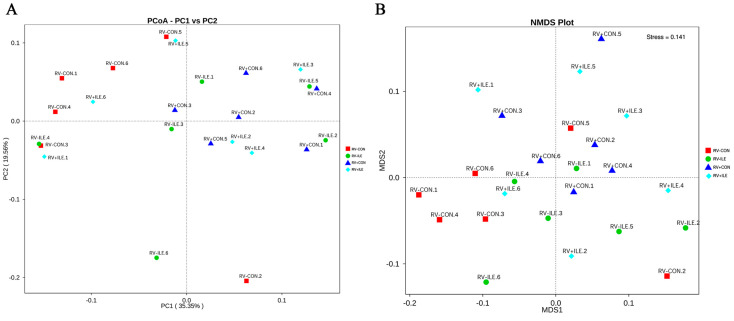
Principal coordinate analysis (**A**) and non-metric multidimensional scaling analysis (**B**).

To assess the impact of isoleucine on the colonic microbiota in RV-infected weaned piglets, the 16S ribosomal RNA (rRNA) gene sequencing was performed. As shown in Table 7, the Chao1, ACE, Shannon, and Simpson indices did not exhibit significant differences among the experimental groups (*p* > 0.05). These results suggested that isoleucine supplementation did not significantly alter the alpha diversity of the colonic microbiota in weaned piglets. In addition, no differences in beta diversity were observed between any of the treatments as indicated by the results of principal coordinate analysis (PCoA) (Figure 4A) and non-metric multidimensional scaling (NMDS) analysis (Figure 4B).

**Figure 5:**
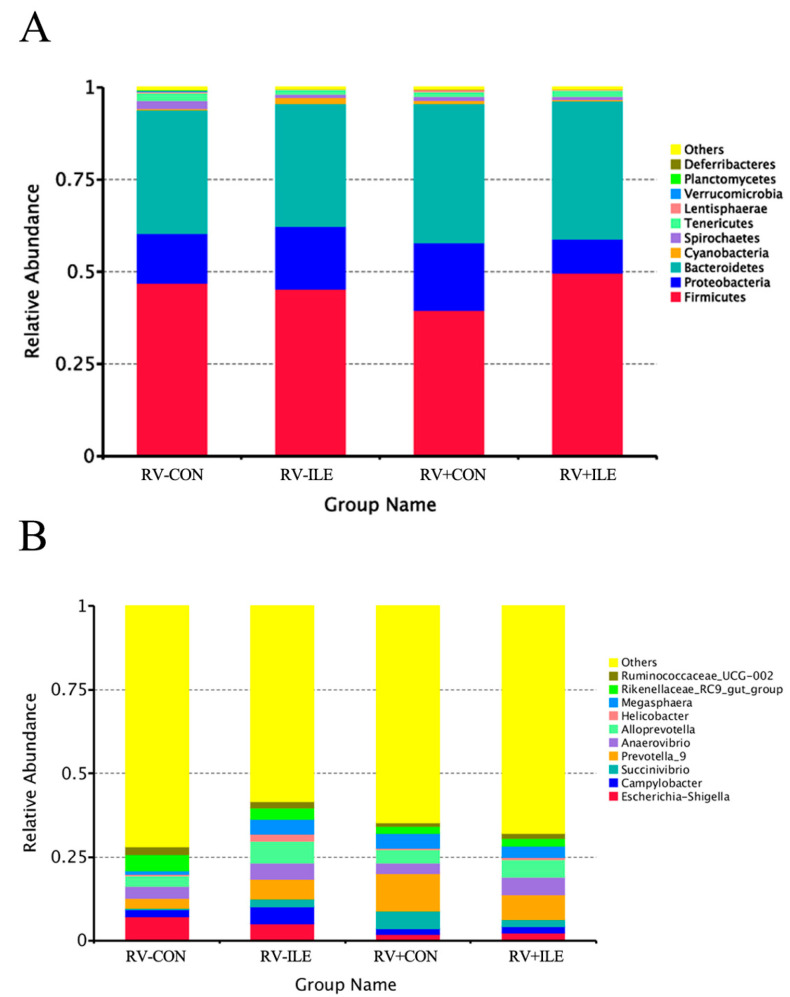
Taxonomy. Phylum-level (**A**) and genus-level (**B**) taxonomic distribution of gut microbiota.

As shown in Figure 5A, at the phylum level, there are a total of 10 dominant bacterial phyla including Firmicutes, Proteobacteria, Bacteroidetes, Cyanobacteria, Spirochaetes, Tenericutes, Lentisphaerae, Verrucomicrobia, Planctomycetes, and Deferribacteres. Among these, the most dominant phyla are Firmicutes, Proteobacteria, and Bacteroidetes. The abundance of the Firmicutes in the RV+CON group was lower than that in RV-CON group. However, the abundance of Proteobacteria and Bacteroidetes in the RV+CON group was higher than that in the RV-CON group. Additionally, the abundance of Firmicutes, Proteobacteria, and Bacteroidetes in the RV+Ile group was similar to that in the RV-CON group. These results suggest that the RV infection in weaned piglets alters the microbial abundance in the colon, while Ile can partially reverse this change.

The genus-level composition of the colonic microbiota in weaned piglets is presented in Figure 5B. The dominant genera include Escherichia-Shigella, Campylobacter, Succinivibrio, Prevotella_9, Anaerovibrio, Alloprevotella, Helicobacter, Megasphaera, Rikenellaceae_RC9_gut_group, and Ruminococcaceae_UCG-002. Compared with the RV-CON group, the RV+CON group showed a significant increase in the relative abundance of Prevotella_9 (*p* < 0.05). However, incorporating Ile into the diet partially mitigated this alteration in RV-infected piglets. Collectively, these results suggested that Ile can somewhat restore the changes in colonic microbiota induced by RV infection in piglets.

### 3.5. Colonic Metabolomics

**Figure 6:**
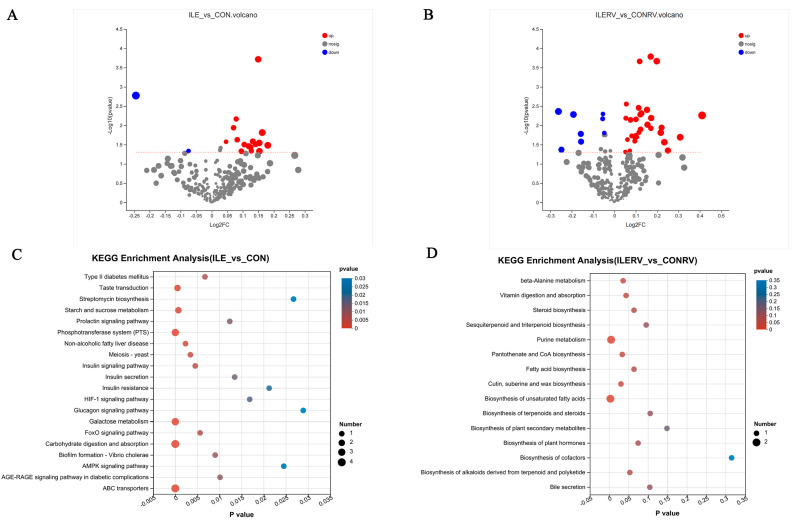
Bioinformatics analysis of the metabolomics. (**A**) The volcano plot of differential metabolites between the RV-ILE group and the RV-CON group. (**B**) The volcano plot of differential metabolites between the RV+ILE group and the RV+CON group. (**C**) KEGG analysis of the differential metabolites between the RV-ILE group and the RV-CON group. (**D**) KEGG analysis of the differential metabolites between the RV+ILE group and the RV+CON group.

To investigate the influence of isoleucine on colonic metabolism in RV-infected weaned piglets, metabolomics analysis was performed. The results indicated that a total of 18 significantly differential metabolites were identified, including 2 significantly downregulated and 16 significantly upregulated metabolites in the RV-Ile group compared with the RV-CON group (Figure 6A). The two significantly downregulated metabolites were triethanolamine and 1-octylsilatrane. The 16 significantly upregulated metabolites included glutaric acid, ferulic acid, maltotriose, sucrose, lactose, D-glucose, and others (Table A1). In addition, a total of 41 significantly differential metabolites were identified in the RV+Ile group compared with the RV+CON group, which included 8 significantly downregulated and 33 significantly upregulated metabolites (Figure 6B). The differentially expressed metabolites are listed in Table A2.

Using the Kyoto Encyclopedia of Genes and Genomes (KEGG) reference pathway database, several metabolic pathways were identified for the differential metabolites in the RV-Ile group versus the RV-CON group. These pathways included starch and sucrose metabolism, galactose metabolism, phosphotransferase system (PTS), carbohydrate digestion and absorption, ABC transporters (Figure 6C). In addition, KEGG pathway enrichment analysis for the RV-infected groups (RV+ILE vs. RV+CON) revealed some differences in metabolic pathways compared with the RV-uninfected groups. The primary enriched metabolic pathways in RV-infected groups included beta-alanine metabolism, vitamin digestion and absorption, steroid biosynthesis, purine metabolism, pantothenate and CoA biosynthesis, biosynthesis of unsaturated fatty acids, fatty acid biosynthesis, biosynthesis of alkaloids derived from terpenoids and polyketides (Figure 6D). These results suggested that the metabolic pattern of nutrients in the colon of weaned piglets is altered following RV infection.

## 4. Discussion

The intestine is responsible for the digestion and absorption of nutrients, while its barrier function helps resist the invasion of pathogenic microorganisms, including viruses, bacteria and others [22,23]. The intestinal immune barrier comprises lymphocytes contained in the intestinal lamina propria, intraepithelial lymphocytes (IELs), and secretory immunoglobulin A (sIgA) secreted by some lymph nodes and plasma cells [24]. The stability of the intestinal barrier function relies on various cytokines present within it, which, together with immunoglobulins, constitute the humoral immunity of the intestine [22]. IL-2 is the first cytokine to be molecularly cloned and has been proven to be essential for T cell proliferation and the generation of effector and memory cells [25]. IL-4 inhibits the inflammatory response through suppressing the differentiation of T helper 1 (Th1) cells and reducing the production of pro-inflammatory factors such as IFN-γ [26]. IL-10 is an anti-inflammatory cytokine that plays a crucial role in limiting the immune response to pathogens, thereby preventing damage to the host [27]. IL-12 is a heterodimeric proinflammatory cytokine that induces the production of interferon-gamma (IFN-γ) by promoting the differentiation of Th1 cells [28,29]. IL-18 is also a pro-inflammatory cytokine that stimulates Th1 cells to produce abundant IFN-γ [30]. IL-22 primarily targets non-hematopoietic epithelial and stromal cells, promoting proliferation and playing a role in tissue regeneration. Additionally, IL-22 regulates host defense at barrier surfaces and is implicated in diseases involving inflammatory tissue pathology [31]. TNF-α (Tumor Necrosis Factor-alpha) is involved in maintaining the immune system’s homeostasis, regulating inflammation, and participating in pathological processes such as chronic inflammation [32,33]. sIgA, as an important component of the immune barrier, plays a crucial role in humoral immunity and is vital for the overall immune competence of the body [34,35]. In this study, we found that Ile did not affect the concentration of pro-inflammatory cytokines including IL-2, IL-12, IL-18, IL-22, and TNF-α in the colon of RV-infected piglets. However, when compared to the RV-CON group, the addition of Ile significantly raised the levels of IL-4, IL-10, and sIgA in the colons of RV-infected piglets. These findings suggest that a diet supplemented with 1% Ile can reduce intestinal inflammation caused by RV infection in piglets. Furthermore, this supplementation also appears to enhance the immune response in RV-infected piglets.

Tight junctions are formed by various transmembrane proteins that connect epithelial cells. Intestinal tight junction proteins, including Zonula Occludens-1 (ZO-1), Occludin, and Claudin-3, are crucial for establishing and maintaining the integrity and functionality of the intestinal mechanical barrier [36,37]. Transmembrane glycoprotein mucin-1 (MUC-1), a member of the mucin family, is secreted by goblet cells and serves as a lubricant, moisturizer, and chemical barrier in normal cells [38]. Lin found that Ile deficiency down-regulated the mRNA expressions of claudin-3, claudin-b, claudin-c, occludin and ZO-1, leading to the intercellular structure damage of fish gills [39]. In this study, we found that inclusion of 1% isoleucine (Ile) significantly elevated the levels of Claudin-3, Occludin, ZO-1, and MUC-1 in the colons of RV-infected piglets compared with the RV+CON group. These findings suggested that isoleucine can promote the colonic intestinal barrier function of RV-infected piglets through promoting the expression of Claudin-3, Occludin, ZO-1, and MUC-1. However, the detailed mechanism requires further investigation.

Firmicutes, Bacteroidetes, Proteobacteria, and Actinobacteria are the dominant phyla in the intestines of healthy piglets [40,41]. Previous study had shown that the abundance of Proteobacteria increases in the intestinal tract of enteritis piglets [42]. Additionally, in diarrheal piglets, the relative abundance of Proteobacteria decreased, while the relative abundance of Sutterella and Campylobacter increased significantly [43]. These results are consistent with our study. We found that Firmicutes, Bacteroidetes, and Proteobacteria were the dominant phyla in piglets, with a notable increase in the relative abundance of Proteobacteria and a decrease in Firmicutes in RV-infected piglets. When 1% Ile was added to the diet, the abundance of Proteobacteria in the colon of RV-infected piglets significantly reduced, while the abundance of Firmicutes increased. In piglets with diarrhea, there was a decrease in the relative abundance of members of the Firmicutes phylum, including Lactobacillus, Enterococcus, Streptococcus, and Clostridium, which are stable members of the normal intestinal microbiota in piglets [44]. Several members of Firmicutes phylum are known to produce short-chain fatty acids and regulate systemic immune responses [45]. Therefore, our study suggests that Ile may promote the recovery of gut microbiota in RV-infected piglets.

Metabolomics analysis revealed that the significantly altered metabolites between the RV+Ile and RV+CON groups were primarily associated with fatty acid biosynthesis, the biosynthesis of unsaturated fatty acids, and purine metabolism. Fatty acids play a crucial role in immune responses and inflammatory processes and influence various cellular signaling and metabolic pathways [46,47]. Unsaturated fatty acids are essential for protecting cells and maintaining their normal physiological functions [48,49]. Purine metabolites provide the energy and cofactors necessary for cell survival and proliferation [50]. RV infection can cause intestinal mucosal damage, and the mechanism by which isoleucine mitigates this damage may involve promoting the synthesis of fatty acids and purine metabolites, thereby protecting normal cells. The decline in intestinal barrier function caused by RV infection, along with the partial alleviation observed with isoleucine supplementation, may be related to the significant upregulation of metabolites involved in fatty acid biosynthesis, the biosynthesis of unsaturated fatty acids, and purine metabolism.

## 5. Conclusions

In conclusion, the addition of isoleucine could improve the immunity and intestinal barrier of the colon in RV-infected weaned piglets. In addition, isoleucine can partially reverse the changes in colonic microbiota caused by RV infection in piglets. These findings offer valuable insights into the role of isoleucine in the prevention and managing RV infection.

## Figures and Tables

**Table 1:** Experimental design.

Treatment Group	Isoleucine (%)	Infection Treatment
RV-CON	0	5 mL Medium
RV-Ile	1	5 mL Medium
RV+CON	0	5 mL RV
RV+Ile	1	5 mL RV

**Table 2:** The composition and nutritional levels of basal diet (%, air-dry basis).

Iltems	Groups	
	CON	1% Ile
**Ingredients** (%)		
Corn	61.27	60.95
Peeled soybean meal, 49% CP	6	6
Soybean protein concentrate, 65%	10	10
Fish meal, 64.5% CP	5	5
Deproteinized whey powder, 5.5% CP	5	5
Soybean oil	3	3
Corn starch	3.3	3.3
Glucose	2	2
Limestone	0.7	0.7
CaHPO_4_	1	1
NaCl	0.3	0.3
Chloride choline	0.15	0.15
Vitamin premix ^1^	0.03	0.03
Mineral premix ^2^	0.3	0.3
L-lysine·HCL	0.43	0.43
L-threonine	0.08	0.08
L-tryptophan	0.4	0.4
DL-methionine	0.06	0.06
L-valine	0.3	0.3
L-isoleucine	0	1
L-alanine	0.68	0
Total	100	100
**Calculated nutrient levels** (%)		
Crude protein	19.79	19.79
Calcium	0.8	0.8
Total phosphorus	0.65	0.65
Available phosphorus	0.48	0.48
Na	0.21	0.21
Cl	0.33	0.33
Lysine	1.35	1.35
Threonine	0.79	0.79
Tryptophan	0.22	0.22
Methionine	0.39	0.39
Valine	0.86	0.86
Isoleucine	0.72	1.72
Leucine	1.56	1.56
Leucine/Isoleucine	217	91
**Metabolizable energy** (**MJ/kg**)	14.05	14.05

^1^ The vitamin premix provided the following per kg of diets: VA 9000 IU, VD3 3000 IU, VE 20.0 IU, VK3 3.0 mg, VB1 1.5 mg, VB2 4.0 mg, VB6 3.0 mg, VB12 0.2 mg, niacin 30.0 mg, Pantothennic 15.0 mg, folic acid 0.75 mg, biotin 0.1 mg. ^2^ The mineral premix provided the following per kg of diets: Zn 100 mg, Mn 4 mg, Fe 100 mg, Cu 6 mg, I 0.14 mg, Se 0.3 mg.

**Table 3:** The ELISA kits used in this study.

Kits	Supplier	Catalog No.
Interleukin-2 (IL-2)	Shanghai Xinle Biotechnology Co., Ltd.	xl-Em0191
Interleukin-4 (IL-4)		xl-Em0194
Interleukin-10 (IL-10)		xl-Em0201
Interleukin-12 (IL-12)		xl-Em0206
Interleukin-18 (1L-18)		xl-Em0211
Interleukin-22 (IL-22)		xl-Er0217
Tumor necrosis factor-α (TNF-α)		xl-Em0359
Secretory immunoglobulin A (sIgA)		xl-Em1716

**Table 4:** The antibodies used in this study.

Antibodies	Supplier	Catalog No.
β-Actin Rabbit mAb (42 kDa)	ABclonal	AC038
CLDN3 Rabbit pAb (27 kDa)		A11650
MUC1 Rabbit mAb (25 kDa)		A19081
Occludin Rabbit pAb (62 kDa)		A12621
ZO-1 Rabbit pAb (250 kDa)		A0659
HRP-conjugated Goat anti-Rabbit IgG (H+L)		AS014

**Table 5:** Effect of isoleucine on colonic morphology in weaned piglets (μm).

Item	RV−		RV+		*p*-Value		
	CON	1% Ile	CON	1% Ile	Ile	RV	Ile×RV
Crypt depth	292.13 ± 24.12	320.68 ± 9.63	279.90 ± 10.48	315.93 ± 15.77	0.24	0.59	0.57
Muscularis thickness	173 ± 26.20	244.59 ± 26.20	183.31 ± 29.58	224.33 ± 19.28	0.49	0.50	0.06

**Table 6:** Effect of isoleucine on the concentration of cytokines and sIgA in the colon of weaned piglets (pg/g protein).

Item	RV−		RV+		*p*-Value		
	CON	1% ILE	CON	1% ILE	ILE	RV	ILE×RV
IL-2	3.82 ± 0.88	3.55 ± 0.88	3.89 ± 1.11	3.70 ± 0.81	0.74	0.62	0.75
IL-4	0.82 ± 0.21 ^bc^	1.35 ± 0.34 ^a^	0.70 ± 0.11 ^c^	1.24 ± 0.20 ^ab^	<0.05	0.41	0.97
IL-10	4.41 ± 1.74 ^ab^	5.10 ± 0.65 ^a^	2.27 ± 0.30 ^c^	3.37 ± 0.86 ^bc^	0.06	<0.05	0.65
IL-12	6.17 ± 0.57	5.70 ± 0.98	5.71 ± 1.10	5.61 ± 1.37	0.52	0.54	0.68
IL-18	2.79 ± 0.69	3.25 ± 0.80	2.67 ± 0.67	2.64 ± 0.53	0.46	0.23	0.41
IL-22	0.54 ± 0.31	0.72 ± 0.35	0.71 ± 0.11	0.62 ± 0.05	0.76	0.82	0.31
TNF-α	3.74 ± 0.35	3.76 ± 1.27	3.39 ± 0.47	3.12 ± 0.78	0.71	0.18	0.68
sIgA	1.03 ± 0.39 ^ab^	1.31 ± 0.27 ^ab^	0.87 ± 0.11 ^b^	1.45 ± 0.12 ^a^	<0.05	0.92	0.21

Note: ^abc^ Means sharing a similar superscript in a row do not differ (*p* < 0.05).

**Table 7:** Analysis of colonic microbial alpha diversity in weaned piglets.

Item	RV−		RV+		*p*-Value		
	CON	1%ILE	CON	1%ILE	ILE	RV	ILE×RV
Chao1	949.29 ± 74.82	908.95 ± 53.55	969.06 ± 33.90	945.57 ± 77.07	0.22	0.28	0.74
ACE	946.12 ± 73.98	912.54 ± 52.09	968.50 ± 30.52	949.05 ± 74.53	0.30	0.25	0.78
Shannon	6.95 ± 1.21	6.56 ± 0.63	6.85 ± 0.48	6.79 ± 0.77	0.50	0.83	0.62
Simpson	0.96 ± 0.07	0.96 ± 0.03	0.97 ± 0.01	0.97 ± 0.02	0.91	0.42	0.88

## Data Availability

The original contributions presented in this study are included in this article, and further inquiries can be directed to the corresponding authors.

## Appendix A

**Table A1:** Differentially expressed metabolites between the RV-ILE group and the RV-CON group.

Metabolite	Regulate	VIP_plsda	VIP_oplsda	*p*-Value
Glutaric Acid	up	1.76	1.98	0.035
1,5-Diphenylpyrazole	up	1.40	1.53	0.007
2-benzamidopropanoic acid	up	1.14	1.15	0.027
2-[4-(carboxymethyl)-2,3-dioxoquinoxalin-1-yl] acetic acid	up	1.54	1.51	0.046
Methyl Palmitate	up	1.75	1.93	0.026
Galacturonic Acid	up	1.62	1.76	0.047
3-Indoleacetic Acid	up	2.13	2.47	0.033
heptadecan-1-ol	up	1.96	2.31	0.028
Ferulic Acid	up	1.72	1.90	0.037
3-chloro-2,5,6-trifluoro-4-(4-phenylphenoxy) pyridine	up	1.43	1.65	0.024
1-Amino-4-Hydroxyanthraquinone	up	2.13	2.27	0.000
Maltotriose	up	1.74	1.76	0.031
Sucrose	up	1.65	1.75	0.031
Lactose	up	2.17	2.53	0.015
octadec-9-enoic acid	up	1.95	2.33	0.047
D-Glucose	up	1.54	1.64	0.012
Triethanolamine	down	1.24	1.17	0.046
1-Octylsilatrane	down	3.05	3.25	0.002

## Appendix B

**Table A2:** Differentially expressed metabolites between the RV+ILE group and the RV+CON group.

Metabolite	Regulate	VIP_plsda	VIP_oplsda	*p*-Value
5-(4-iodoanilino)-1,3-thiazolidine-2,4-dione	up	1.18	1.30	0.015
Glycerol 1-Phosphate	up	2.19	2.36	0.020
Hypoxanthine	up	2.89	3.10	0.006
Myristic Acid	up	1.01	1.03	0.049
3-Hydroxycinnamic Acid	up	1.20	1.27	0.020
Pantothenic Acid	up	1.05	1.12	0.023
4-O-Methylphloracetophenone	up	1.00	1.03	0.046
2-methylidene-4-phenylbutanoic acid	up	1.89	1.89	0.011
Phytanic Acid	up	1.44	1.50	0.003
1,3,5-triphenylbenzene	up	1.56	1.64	0.013
4-(1H-indol-3-yl)-9H-carbazole-1-carboxylic acid	up	1.22	1.29	0.003
5-[di(propan-2-yl)amino]-2-pyridin-3-ylpent-3-yn-2-ol	up	1.93	2.26	0.027
5-Hydroxyindole-2-Carboxylic Acid	up	1.34	1.26	0.018
diethyl-hexadecoxy-[(2,3,4,5,6-pentafluorophenyl)methoxy]silane	up	1.30	1.23	0.020
D-Glucose-6-Phosphate	up	1.71	1.96	0.045
Nonadecanoic Acid	up	1.53	1.64	0.004
decanedioic acid	up	1.18	1.27	0.006
dicyclohexyl(4-dicyclohexylphosphanylbutyl)phosphane	up	1.35	1.42	0.020
Arachidic Acid	up	1.34	1.47	0.007
Uric Acid	up	1.34	1.46	0.007
henicosanoic acid	up	1.60	1.67	0.000
1-Stearoyl-Rac-Glycerol	up	1.79	2.07	0.004
Docosenoic Acid	up	2.03	2.21	0.000
Behenic Acid	up	1.67	1.87	0.005
2-[4-[N-[1-[4-(carboxymethoxy)phenyl]ethylideneamino]-C-methylcarbonimidoyl]phenoxy]acetic acid	up	1.70	1.90	0.005
4-hydroxy-9,10-dioxoanthracene-2-carboxylic acid	up	1.76	2.03	0.006
methanesulfonyl chloride	up	1.98	2.24	0.015
diethyl-hexadecoxy-(4-methylcyclohexyl)oxysilane	up	1.74	2.02	0.010
2-(2,4b-dimethyl-2,3,4,4a,5,6,7,8,8a,9,10,10a-dodecahydro-1H-phenanthren-1-yl)acetonitrile	up	1.43	1.37	0.025
Squalene	up	1.91	2.07	0.000
Coprostan-3-One	up	1.47	1.63	0.007
7-methoxy-2-(4-methoxyphenyl)-4H-thieno[2,3-b]indole	up	1.17	1.23	0.019
5-(4-aminophenyl)-4-(3-iodophenyl)-1,3-thiazol-2-amine	up	1.68	1.53	0.012
(N-hydroxy-N’-phenylcarbamimidoyl) benzenecarboximidate	down	0.95	1.06	0.016
10,14-dioxapentacyclo[11.7.0.03,11.04,9.015,20]icosa-1(13),2,4,6,8,11,15,17,19-nonaene	down	1.05	1.06	0.005
Octamethylcyclotetrasiloxane	down	1.72	1.90	0.043
heptadecan-1-ol	down	1.65	1.93	0.017
2,4-bis(4-methylphenyl)-6-phenyl-1,3,5-triazine	down	1.83	1.89	0.026
2-methylthieno[2,3-e]thiazine 1,1-dioxide	down	2.18	2.43	0.004
hexadecoxy-dimethyl-pentoxysilane	down	1.12	1.25	0.007
octadec-9-enoic acid	down	2.00	2.28	0.005
